# A Retrospective Study on Dietary FODMAP Intake in Celiac Patients Following a Gluten-Free Diet

**DOI:** 10.3390/nu10111769

**Published:** 2018-11-15

**Authors:** Leda Roncoroni, Luca Elli, Luisa Doneda, Karla A. Bascuñán, Maurizio Vecchi, Federico Morreale, Alice Scricciolo, Vincenza Lombardo, Nicoletta Pellegrini

**Affiliations:** 1 Center for Prevention and Diagnosis of Celiac Disease, Gastroenterology and Endoscopy Unit, Fondazione IRCCS Ca’ Granda, Ospedale Maggiore Policlinico, 20122 Milan, Italy; lucelli@yahoo.it (L.E.); kbascunan@med.uchile.cl (K.A.B.); scricciolo.alice@gmail.com (A.S.); vicky.l@hotmail.it (V.L.); 2 Department of Biomedical, Surgical and Dental Sciences, University of Milan, 20131 Milan, Italy; luisa.doneda@unimi.it; 3 Department of Nutrition, University of Chile, 8380453 Santiago, Chile; 4 Department of Pathophysiology and Transplantation, University of Milan, 20122 Milan, Italy; maurizio.vecchi@policlinico.mi.it; 5 Gastroenterology and Endoscopic Unit, Fondazione IRCCS Ca’ Granda, Ospedale Maggiore Policlinico, 20122 Milan, Italy; 6 Human Nutrition Unit, Department of Food and Drug, University of Parma, 43121 Parma, Italy; morreale.federico@gmail.com (F.M.); nicoletta.pellegrini@unipr.it (N.P.)

**Keywords:** FODMAP intake, celiac disease, irritable bowel syndrome, gluten-free diet, gastrointestinal symptoms

## Abstract

Our aim was to evaluate the intake of foods containing fermentable oligo/di/mono-saccharides and polyols (FODMAP) as a possible factor that induces gastrointestinal symptoms in treated celiac disease (CD) patients. We collected seven-day weighed food records for 104 CD patients and 91 healthy volunteers. All evaluated food items were from sources with high and low content of FODMAP, which were divided into cereals and sweets, sweeteners and soft drinks, fruits, dried fruits, and vegetables. Nutrient intake was calculated using the food database of the European Institute of Oncology. The symptoms reported were assessed by a Rome IV Irritable bowel syndrome (IBS) diagnostic questionnaire and by specific questions for the evaluation of functional gastrointestinal disorders (FGIDs). The 12% of CD patients met IBS symptoms criteria as opposed to 6% of controls (*p* = 0.09) and 27% of patients reported FGIDs symptoms vs. 22% of healthy controls (*p* = 0.42). The intake by CD patients was significantly higher than healthy volunteers for: sweeteners and sugars with low content of FODMAP (*p* = 0.0007), fruits, dried fruits, and vegetables high in FODMAP (*p* = 0.003) and low in FODMAP (*p* = 0.04) when compared to controls. CD patients had a lower intake of cereals and sweets with a high content of FODMAP (*p* = 0.00001). Healthy volunteers consumed significantly higher alcoholic beverages and fats high in FODMAP (both *p* < 0.044). The mean daily intake of other food categories did not differ between both groups. Even though CD patients had a low intake of gluten-free cereals high in FODMAP, they still consumed a significant amount of fruits and vegetables high in FODMAP. The clinical effect of a concomitant gluten-free diet and low-FODMAP diet should be prospectively evaluated as a supportive therapy in CD patients.

## 1. Introduction

Celiac disease (CD) is a chronic autoimmune enteropathy triggered by dietary exposure to gluten and is characterized by villous atrophy. CD is considered the most common chronic enteropathy in Western countries with an estimated prevalence ranging from 0.5 to 1% [1,2]. An altered T cell mediated immune response triggered by gluten has a central role in the pathogenesis of CD. Genetic predisposition plays a key role in the development of CD and the presence of the HLA DQ2 and/or DQ8 haplotypes is a necessary (but not sufficient) factor [3,4]. CD patients may present with gastrointestinal symptoms, extra-intestinal symptoms, or no symptoms at all. The classical clinical symptoms include diarrhea, steatorrhea, and weight loss due to malabsorption [5]. 

Gluten is a complex of high molecular-weight proteins found in the endosperm of grass-borne grains including wheat, barley, and rye [6]. The treatment of CD is the withdrawal of gluten from the diet (i.e., a gluten-free diet, GFD), which usually silences symptoms and normalizes serological and histological signatures. CD is considered a lifelong disorder and, if left untreated, is associated with increased morbidity and mortality [7,8]. Despite the strict adherence to a GFD, there is a group of CD patients who keep suffering from functional gastrointestinal disorders (FGIDs) [9]. FGIDs include irritable bowel syndrome (IBS) and non-specific gastrointestinal symptoms (pain, diarrhea, bloating, nausea, vomiting). IBS is currently identified to be a global problem with a prevalence of 11%, which specifically affects women as compared to men (14.0% vs. 8.9%, respectively) [10,11]. IBS is characterized by a multiplicity of clinical symptoms such as abdominal pain, bloating, and changes in bowel habits including constipation and/or diarrhea. It also compromises the patients’ abilities and quality of life, which implicates an increase in healthcare provision costs, use of drugs, and absence/leave from work [12].

The criteria for diagnosing gastrointestinal disorders have been revised by the Rome Foundation in their latest update (Rome IV, 2016). The pathogenesis of IBS is multi-factorial and may be connected to visceral hyper-sensitivity, alterations of the gut-brain axis and the microbiota, post infectious consequences, and environmental and genetic factors [13]. IBS can be suspected in patients suffering from persistent abdominal pain associated with more than one condition: defecation, change in the frequency of defecation, and change of stool form [14]. Moreover, IBS can be classified as IBS-C (predominant constipation), IBS-D (predominant diarrhea), and IBS-M (mixed) [15]. Nowadays medication (antispasmodics, bulking agents, probiotics, laxatives, or anti-depressants), diet, and lifestyle adjustments have been the main therapeutic options for IBS [16]. 

In recent years, data supporting that the dietary restriction of fermentable oligo/di/mono-saccharides and polyols (FODMAP) for the management of IBS symptoms have emerged [17,18,19]. Studies evaluating a low-FODMAP diet in patients with IBS have consistently shown symptomatic benefits in the majority of patients [20]. However, one study that compared the diet commonly used in these patients vs. a low-FODMAP diet failed to demonstrate differences in the improvement of the symptomatology [21]. The use of the low-FODMAP diet has been evaluated in other digestive pathologies with contradictory results. Pedersen et al. showed some degree of improvement in functional gut symptoms of patients with inflammatory bowel disease (IBD) [22] while Cox et al. showed exacerbated functional gastrointestinal symptoms in patients with IBD [23]. On the other hand, Testa et al. were able to demonstrate that a low-FODMAP diet could be one option to counteract abdominal symptoms in patients with IBS, non-active IBD, or CD on a GFD, which improves their quality of life [24].

The main dietary sources of FODMAP include fructose in honey, apples, and pears, fructans in wheat, rye, onion, and garlic, galactans in cabbage and legumes, lactose in milk and dairy products, and polyols including sorbitol and mannitol in stone fruits, mushrooms, and artificial sweeteners [25]. Among FODMAP, fructans and galacto-oligosaccharides are considered prebiotics. Prebiotics are non-digestible selectively fermented dietary fibers that promote the growth of positive bacterial genera in the gastrointestinal tract. Prebiotics such as inulin-type provides health benefits to the host [26]. These non-digestible food ingredients are also extensively employed in the food industry [27]. The main feature of these carbohydrates is their poor absorption in the small bowel. This is due to different reasons including reduced or absent digestive enzymes or by their slow transport across the intestinal mucosa. Undigested and unabsorbed FODMAP have the peculiarity to be highly osmotic and rapidly fermented by bacteria in the gut, which causes distension, bloating, cramping, and diarrhea, which are all symptoms found in IBS [28]. Moreover, high concentrations of FODMAP in the ileum and proximal colon exert osmotic pressure, which draws greater amounts of water into the lumen [29]. Fermentation as produced by short-chain FODMAP intensifies the luminal H_2_ and CH_4_ production and causes pain in the lumen. This gas production can be measured with a breath test, which shows an increase between 0–5 h after the ingestion of FODMAP [30]. As proposed mechanisms, it has been recently shown as a link between FODMAP and inflammation induction in vivo [31]. Zhou et al. [32] reported that a high-FODMAP diet could increase the serum lipopolysaccharide level and intestinal inflammation together with visceral hypersensitivity. On the contrary, a diet low in FODMAP could reduce LPS levels and the inflammatory status.

Nowadays, it is of great interest to evaluate if persistent gastrointestinal symptoms in celiac patients, classified as FGIDs and IBS, can be provoked by the ingestion of FODMAP. We aimed to evaluate the intake of foods containing FODMAP in a group of patients with CD compared with the intake of healthy volunteers.

## 2. Materials and Methods

### 2.1. Patients

The participants were recruited among patients referred to the Center of Prevention and Diagnosis of Celiac Disease Fondazione IRCCS Ca’ Granda Ospedale Maggiore Policlinico, Milan, Italy. All the subjects were recruited between October 2012 and August 2014 and the data were collected during the same period. Over that time, during their annual medical examination, the patients were screened for their adherence to GFD by using the celiac diet adherence test (CDAT) [33].

The enrolment process of CD patients and healthy individuals has been previously described [34,35]. All patients included in the study fulfilled the Rome IV questionnaire criteria for IBS, functional dyspepsia, and functional bloating [15]. In detail, each patient was asked if they have had recurrent abdominal pain arising for at least 6 months and being present at least once a week in the last 3 months, which is associated with changes in the stool’s volume and consistency. The presence of diarrhea and/or constipation associated with abdominal pain was confirmed in case of presence of IBS. They were also asked for the presence of dyspepsia and/or any other functional symptoms such as bloating (for FGIDs). The exclusion criteria were: CD diagnosis less than two years, the presence of metabolic or chronic disease (e.g., diabetes mellitus, Crohn’s Disease, cardiovascular and neuro-vascular disease, cancer, neuro-degenerative disease, and rheumatoid arthritis), pregnancy or lactation, vegetarianism, or veganism. A group of healthy volunteers was recruited among students, researchers, and professors of the Universities of Parma, Parma, and University of the Studies, Milan. For healthy volunteers, CD was excluded by means of serological tests (anti-tissue transglutaminase IgA). Exclusion criteria for the healthy participants were the same as for the CD patients except for the diagnosis of CD.

The final sample included 104 celiac patients and 91 healthy controls. All the participants who agreed to participate signed a written informed consent and were enrolled in the study. The local Ethics Committee for Human Research of the City of Milan approved the protocol. The study was registered at ClinicalTrials.gov (ID NCT01975155).

### 2.2. Dietary Records

The total food and beverage consumption was assessed by using a food diary filled in daily for a total of seven days, which was previously described [36]. The participants were trained on how to record all of the food consumed by a nutritionist. The participants were asked to send their completed seven-day weighed food record to the Department of Food and Drugs of the University of Parma. A nutritionist reviewed the diaries and, in case of any error or omission, the participants were contacted by phone to clarify the issues.

The nutrient intake was calculated by using a Microsoft Access application (version 2003, Microsoft Corp, Redmond, WA, USA) linked to the European Institute of Oncology’s food database, which covered the nutrient composition of 900+ Italian foods [37], integrated with the nutrient composition of 60 gluten-free foods available in the Italian market [38]. When a food recorded in a participant’s seven-day weighed food record could not be found in the database, an alternative food was appropriately chosen based on similarities in energy and nutrient composition. The output consisted of the mean daily intake of energy and food items for each individual.

Food items of interest for the FODMAP intake evaluation were grouped into the following food categories (a summary of food sources of FODMAP, as reported in the literature, is provided in Table 1): pasta, bread (including crackers and salty snacks), other cereals (including corn, quinoa, buckwheat, and rice), fruits, vegetables and legumes, sweeteners (honey, saccharin, fructose, barley malt syrup), and sweets (including biscuits, sweet snacks, breakfast cereals, ice cream, candies, and chocolate), dried fruits, oil and fats, dairy products (including milk, yogurt, cream, cheese), soft drinks, juices, coffee/tea, and alcoholic beverages [18]. The categories of foods high and low in FODMAP content were also grouped in larger or macro-categories: cereals and sweets, sweeteners and soft drinks, fruits, dried fruits, and vegetables. For each individual, the mean daily intake of each food macro-category, i.e., low and high-FODMAP contents, was calculated.

### 2.3. Statistical Analysis

Data were accordingly described as mean ± SD or median (with interquartile range), according to variables’ distribution (evaluated by the Shapiro-Wilk test). The Pearson chi-square test was used to compare the number of individuals reporting IBS and FGIDs in between both studied groups. The daily nutrient intake data of energy, water, and FODMAP-containing foods were computed and tabulated. To reduce the effect of implausible extreme values on the analysis, we checked that the ratio of total energy intake determined from the food record to the basal metabolic rate (determined by the Harris-Benedict equation) of each participant was not in the first or the last percentile of the distribution. The dietary data were compared between the control patients and the celiac disease patients by using the independent sample Student’s *t*-test or the Wilcoxon rank-sum test. STATA^®^ v. 13.1 (StataCorp, College Station, TX, USA) was used in all the analyses. Statistical significance was set at a 5% α-level.

## 3. Results

### Patients

**Figure 1:**
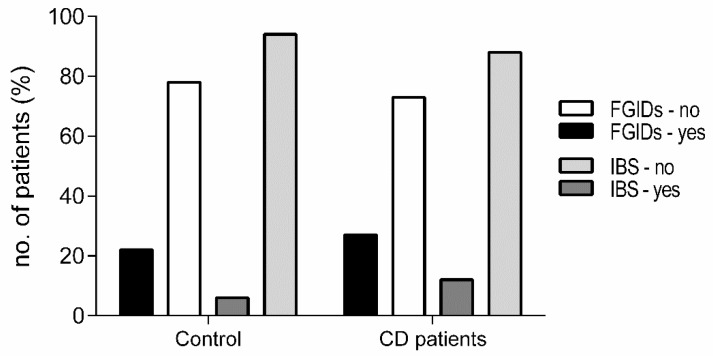
Presence of gastrointestinal symptoms. Data are shown as a percentage of individuals reporting IBS and FGIDs symptoms between both studied groups. IBS: Irritable bowel syndrome, FGIDs: Functional gastrointestinal disorders, CD: Celiac disease.

Patients in the CD group were older (*p* = 0.0002) and there was a higher percentage of females in both groups (82.7% and 68.1% of CD patients and controls, respectively). Both groups were comparable regarding their mean BMI (22.4 ± 3.3 and 22.2 ± 3.0 for the CD and control group, respectively) and daily energy intake (2054.8 kcal (397.3) and 2044.7 kcal (329.3)), respectively. When analyzing the prevalence of gastrointestinal symptoms in both groups, we found out that 12% of CD patients suffered from IBS when compared with 6% of the controls (*p* = 0.09) while 27% of CD patients suffered from FGIDs symptoms (5.77% functional dyspepsia, 10.58% functional bloating) against 22% of controls (*p* = 0.42). Approximately 10.5% of CD patients did not suffer from either IBS, functional dyspepsia, or functional bloating (Figure 1).

Regarding the dietary intake, a slightly higher total food intake was observed in the CD group, but the energy and water intake were similar between both groups (Table 2). A higher intake of foods with high FODMAP content was observed in the controls (*p* < 0.001) while a higher intake of foods with low FODMAP content was observed in CD patients (*p* < 0.001). 

The analysis of FODMAP-containing food intake according to food categories is shown in Table 3 and Table 4. The consumption of high-FODMAP foods was higher in the control group with the exception of fruits, which was higher in the CD group. In turn, low-FODMAP food consumption was greater in the CD group. A larger amount of high-FODMAP cereals (*p* = 0.0001), high-FODMAP sweets (*p* = 0.0001), high-FODMAP alcoholic beverages (*p* = 0.0001), and high-FODMAP fats (*p* = 0.0447) was observed in the control group when compared to celiac patients. Instead, the CD group consumed significantly higher amount of cereals with low FODMAP content (*p* = 0.0001), low-FODMAP sweets (*p* = 0.0001), low-FODMAP sweeteners (*p* = 0.0099), fruit both high and low in FODMAPs (respectively *p* = 0.001 and *p* = 0.0158), and low-FODMAP juices (*p* = 0.0228). The mean daily intake of the other food categories did not differ between the two groups (Table 3).

When the foods containing FODMAP were grouped into large categories (i.e., cereals and sweets, sweeteners and sugars, fruits, dried fruits, and vegetables), a higher consumption of cereals and sweets with high FODMAP content was present in the control group (*p* = 0.00001) while CD patients showed significantly higher consumption of fruits, dried fruits, and vegetables with high FODMAP content (*p* = 0.003). In addition, other large categories of foods with low FODMAP content were significantly more consumed by the CD patients as compared to the control group (Table 4). Lastly, no correlation between FODMAP intake and gastrointestinal symptoms was found.

## 4. Discussion

This is the first time that a classification is made in relation to food consumption, according to the amount of FODMAP in patients with CD. In our study, 12% of CD patients met the Rome IV symptom criteria and 27% suffered from FGIDs symptoms. The data available confirms that the prevalence of IBS-type symptoms among the group of celiac patients is higher than that in a group of control subjects [39]. While it was true, CD patients showed a low intake of high FODMAP cereals, which reflects treatment with a GFD. We observed that the intake of high FODMAP fruits in this group could in some way be associated with the higher percentage of CD patients reporting IBS and FGIDs symptoms as compared to controls [40]. 

When CD is diagnosed, there is usually the assumption that gastrointestinal symptoms will resolve once gluten is eliminated from the diet. Instead, it has been demonstrated that some celiac patients carry on suffering of gastrointestinal symptoms one year after diagnosis even with their correct adherence to GFD and normalization of serum tTG levels [9]. Such a finding also includes the fact that celiac patients do not report great quality-of-life scores as compared to those of the healthy population [41]. Therefore, the hypothesis that only mucosal inflammation may have a sensitizing effect or predispose to IBS-type symptoms is not the only way to run. The diet can play a pivotal role in the induction of IBS symptoms [42] especially FODMAP [43]. IBS is a common syndrome characterized by abdominal discomfort or pain and is associated with altered bowel habits [14]. Currently, once major organic gastrointestinal disorders have been excluded, specialists focus on the possible link between an “IBS” clinical picture and molecules such as α-amylase/trypsin inhibitors (resistance molecules contained in cereals to fend off pests and parasites) or dietary habits such as the intake of lactose, dietary nickel, poorly absorbed, and short-chain carbohydrates (i.e., FODMAP). FODMAP are contained in different types of cereals such as wheat, barley, rye, and derived products and sweets and sweeteners such as honey, saccharin, and fructose. There are two other categories that contain high levels of FODMAP including several types of fruits/dried fruit (such as apple, apricot, peach, pear, watermelon, and plum) and vegetables (such as artichoke, asparagus, cauliflower, onion, garlic, and beans). In addition, dairy products are given attention for their high FODMAP content (lactose): milk, yogurt, fresh cheese, semi-aged cheese, and aged cheese. At present, no data is available about FODMAP intake in CD patients [44,45].

In our study, a group of celiac patients was compared with a group of healthy subjects with regard to their intake of FODMAP through foods containing different (low and high) levels of them. We recorded all the foods and beverages consumed during a week and then consumed foods that were classified as being high or low in FODMAP content. We used such an approach since the direct measurement of FODMAP content by means of reliable analytical methods in common foods is unavailable yet. As expected, CD patients had a lower intake of cereals and sweets containing high FODMAP levels (including the gluten-containing grains forbidden to celiac patients) as compared to healthy volunteers. Conversely, patients had a higher intake of low FODMAP cereals including cereals allowed in GFD such as rice, corn, and pseudo cereals. Interestingly, a significantly higher intake of fruits (both high and low in FODMAP) was found in the CD patients. Moreover, when all vegetable foods, i.e., fruits, dried fruits, and vegetables were grouped together, we observed a significantly high intake of both categories in CD patients when compared to healthy volunteers. This suggests a greater level of attention about a healthy diet paid by CD patients than controls. However, since cereals contain fructans, fruit and vegetables are two food categories with a high content of FODMAP. This high intake of fruits rich in FODMAP might partly explain the tendency to a higher number of CD patients reporting IBS and FGIDs symptoms when compared to controls. When comparing sweeteners intake, both groups had a comparable low intake of high-FODMAP foods. This suggests that fructose as a sweetener was not commonly used by all the volunteers. In fact, only 12 out of 91 controls and 11 out of 104 CD patients made use of it (data not shown). By contrast, CD patients consumed more low-FODMAP sweeteners such as sugar and aspartame than controls. Lastly, the results suggest that patients with CD did not exclude or limit dairy products with a high content of FODMAP, which shows a pattern similar to that of the control group. Of note, milk and its derivatives provide important nutrients due to its content of vitamins, minerals, and macronutrients essential for the growth, development, and maintenance of tissues [46]. On the other hand, the benefits of the fatty component of dairy products have been demonstrated and elicit a greater bioavailability of high-value nutrients and also show anti-inflammatory properties [47,48,49]. The presumed anti-inflammatory benefits described in dairy products could potentially be the reason, at least in part, why some CD patients can tolerate them despite the secondary lactose intolerance described in some of them.

Currently, little is known about the level of FODMAP intake in different population groups. Nevertheless, it is of great interest to confirm a potential relationship between specific dietary carbohydrates and gastrointestinal symptoms [25]. FODMAP have been reported as triggers of gastrointestinal symptoms in IBS, Crohn’s disease, and ulcerative colitis with the hypothesis being that the rapid fermentation and passage of FODMAP through the gastrointestinal tract leads to an increase in intestinal permeability [50]. Particularly in IBD patients who have superimposed IBS, the luminal distension (caused by the fermentation of FODMAP by bacteria in the distal small and proximal large bowel) can cause gastrointestinal symptoms such as bloating, distension, cramping, and diarrhea [51]. There are few antecedents on the long-term effectiveness, acceptability, and nutrient adequacy of a diet with low FODMAP content. Studies have evaluated retrospectively and prospectively changes in the gastrointestinal symptomatology [52,53,54] while long-term nutritional characteristics of a diet with low FODMAP content had not been studied. O’Keeffe et al. [55] evaluated the long-term impact of the low FODMAP diet on clinical response, nutritional adequacy, dietary acceptability, and quality of life related to foods. The authors concluded that the education provided in relation to a diet with low FODMAP content is effective in the management of IBS by allowing the maintenance of a nutritionally adequate diet among patients. However, one unintended consequence associated with FODMAP restriction is its impact on the gut microbiota. Probiotic supplementation with Bifidobacterium is associated with a reduction in IBS symptoms but the low FODMAP diet markedly reduces luminal Bifidobacterium concentration [56]. It is likely that both interventions can be successful in specific groups of patients [30]. In this sense, Staudacher et al. [19] showed that a low-FODMAP diet was associated with adequate relief of symptoms in patients with IBS when compared to the placebo and concomitant administration with probiotics (Bifidobacterium) could be administered to restore these bacteria in patients on a low-FODMAP diet.

For the first time, this study evaluated the intake of low or high-FODMAP foods in CD patients affected by IBS and FGIDs symptoms. As limitations, we would like to mention that our results were obtained through food diaries in a seven-day period, which is a period of time that is short and does not allow conclusions about the long-term impact of this dietary pattern. Even though the recording period is short, the participants accurately reported and weighed all the consumed food and beverages. Moreover, in a previous publication on almost the same participants, we compared this tool to a food frequency questionnaire and demonstrated that the two tools were correlated especially in terms of food groups [34]. The study of the diet during a longer period of time could provide relevant knowledge regarding dietary characteristics in patients with CD. Because of the explorative nature of our study, we could not find any causative relationship between the intake of FODMAP-rich foods and the presence of gastrointestinal symptoms in our patients. However, the clinical effect of a GFD together with low-FODMAP diet should be prospectively evaluated as a supportive therapy for CD patients. In this sense, our group has recently reported that a GFD associated with a low-FODMAP content is beneficial as a supportive therapy for a group of CD patients with persistent gastrointestinal symptoms [57]. When consolidated data on the FODMAP content of foods become available, it will allow the investigation of specific FODMAP groups among oligosaccharides, disaccharides, monosaccharides, and polyols that are more consumed by CD patients than by controls.

ClinicalTrials.gov, ref. no. NCT01975155.

## Figures and Tables

**Table 1:** Foods with high and low amounts of FODMAP ^†^.

Food Category	High in FODMAP	Low in FODMAP
Alcoholic beverages	Sweet wine, rum, vodka, whiskey	Beer, red wine, rosè wine, white wine, sparkling wine, gin
Cereals	Wheat, barley, rye, bran, whole wheat flour, spelt, Kamut^®^ (Khorasan wheat)	Quinoa, rice, buckwheat, pearl millet, corn, gluten-free flour, bulgur, sourdough spelt bread, cornflakes, puffed rice
Sweets	Apricot jam, peach jam, blackberry jam, plum jam, milk chocolate	Strawberry jam, peanut butter, lactose-free ice cream, gluten-free sweets
Sweeteners	Honey, saccharin, fructose, malt syrup	Aspartame, sucrose, sugar, brown sugar
Fruit	Apple, apricot, avocado, ripe banana, blackberry, litchi, mango, white peach, yellow peach, pear, persimmon, pomegranate, blackcurrant, cranberry, plum, watermelon, cherry	Banana, blueberry, strawberry, raspberry, melon, white melon, kiwi, orange, mandarin orange, red grapes, black grapes, green grapes, lemon, coconut, pineapple, loquat, papaya, prickly pear, passion fruit, grapefruit
Dried fruit	Raisin, dried date, apple, apricot, fig, mango, pear, plum, cashew nut, pistachio	Hazelnut, pine nut, walnut, flax seed, sunflower seed, dried banana, coconut, dried cranberry, dried coconut
Fats	Butter, margarine, cream, lard	Olive oil, extra-virgin olive oil, sunflower oil, almond oil, peanut oil, linseed oil, sesame oil, rice oil, palm oil, coconut oil, colza oil, soybean oil
Dairy products	Milk, yogurt, fresh cheese, semi-aged cheese, aged cheese	Lactose-free milk, lactose-free yogurt, lactose-free cheese, feta, cottage cheese, Parmesan
Soft drinks	Apple juice, orange juice, apricot juice, pear juice, peach juice, pomegranate juice	Pineapple juice, lemon juice, cranberry juice, vegetables juice, orange fresh-squeezed juice
Vegetables	Red radicchio, green radicchio, artichoke, asparagus, cauliflower, red cabbage, green cabbage, sauerkraut, pea, snow pea, mushroom, garlic, leek, onion, Jerusalem artichoke, bean, white bean, lupine bean, broad bean (Vicia fabai), chickpea, broccoli, beet	Pepper, carrot, eggplant, zucchini, potato, sweet potato, tomato, lettuce, fennel, fennel leaves, spinach, chard, radish, green bean, turnip, chicory, rocket, stick, watercress, endive, valerian, olive, sweet corn, chestnut, pumpkin flower, cucumber, chives, chili pepper, ginger, Brussels cabbage, soy sprout, bean sprout, healing herb, okra, truffle, green alga, celery, pumpkin

^†^ Examples of foods classified as having the high and low amount of FODMAP. FODMAP: fermentable oligo/di/mono-saccharides and polyols.

**Table 2:** Daily dietary intake of celiac patients and healthy controls.

Dietary Intake	Healthy Controls (*n* = 91)	Celiac Patients (*n* = 104)	*p* Value ^†^
Food intake, g/day	1569.5 ± 337.0	1656.5 ± 284.2	0.05 ^‡^
Energy intake, kcal/day	2054.8 (397.3)	2044.7 (329.3)	0.68
Water intake, mL/day	742.8 (400.0)	647.1 (452.6)	0.52
High-FODMAP, g/day	681.4 (261.0)	457.4 (208.4)	0.00001
Low-FODMAP, g/day	835.1 (364.8)	1119.6 (366.9)	0.00001
High-FODMAP, %	45 (10)	28 (10)	0.00001
Low-FODMAP, %	54 (10)	71 (10)	0.00001

Data as median values (IQR) unless otherwise is indicated. a data as mean ± SD. ^†^ *p* value for the Wilcoxon rank-sum test unless otherwise is indicated. ^‡^ *p* value for the *t*-test for two independent groups.

**Table 3:** Daily intake of food categories containing low and high amounts of FODMAP by celiac patients and healthy controls.

Food Categories (g/day)	Healthy Controls		Celiac Patients		*p* Value *	*p* Value **
	High FODMAP	Low FODMAP	High FODMAP	Low FODMAP		
Alcoholic beverages, g/day	5.7 (57.1)	21.4 (64.2)	0 (5.7)	6.7 (61.1)	0.0001	0.22
Cereals, g/day	228.0 (61.1)	171 (30.7)	0 (0)	222.7 (95.1)	0.0001	0.0001
Sweets, g/day	22.8 (32.1)	2.1 (11.4)	2.8 (9.1)	35.7 (51.6)	0.0001	0.0001
Sweeteners, g/day	0 (1.6)	6.4 (8.8)	0 (1.2)	8.2 (11.7)	0.49	0.0099
Fruits, g/day	45.7 (108.7)	85.7 (115.7)	100.4 (126.8)	116.4 (128.0)	0.001	0.0158
Dried fruits, g/day	0 (0.2)	0 (2.1)	0 (0)	0.5 (4.1)	0.53	0.28
Fats, g/day	3.3 (6.7)	25.7 (14.4)	2.4 (5.6)	26.0 (13.6)	0.0447	0.25
Dairy, g/day	188.6 (211.4)	7.4 (16.4)	196.2 (184.2)	7.5 (17.6)	0.61	0.85
Juices, g/day	1.4 (47.1)	0 (2.8)	25.7 (67.8)	0.7 (34.2)	0.49	0.0228
Vegetables, g/day	55.0 (87.4)	236.2 (146.4)	73.1 (85.8)	245.8 (139.6)	0.21	0.60

Data as median values (IQR). * *p* value for comparisons between groups regarding high-FODMAP foods. ** *p* value for comparisons between groups regarding low-FODMAP foods.

**Table 4:** Daily intake of grouped food categories containing low and high amounts of FODMAP by celiac patients and healthy controls.

Food Categories	Healthy Controls (*n* = 91)		Celiac Patients (*n* = 104)		*p* Value *	*p* Value **
	High FODMAP	Low FODMAP	High FODMAP	Low FODMAP		
Cereals & sweets, g/day	253.7 (74.2)	28.6 (37.4)	3.6 (11.4)	265.8 (103.4)	0.00001	0.00001
Sweeteners & sugar, g/day	14.2 (47.1)	9.0 (14.4)	28.6 (73.6)	15.4 (38.5)	0.64	0.0007
Fruits, dried fruits & vegetables, g/day	135.0 (185.2)	360.7 (175.2)	181.3 (126.0)	399.8 (232.1)	0.003	0.04

Data as median values (IQR). * *p* value for comparisons between groups regarding high-FODMAP foods. ** *p* value for comparisons between groups regarding low-FODMAP foods.

## References

[1] Rubio-Tapia A., Murray J.A. (2010). Celiac disease. Curr. Opin. Gastroenterol..

[2] Van Berge-Henegouwen G.P., Mulder C.J.J. (1993). Pioneer in the gluten free diet: Willem-Karel Dicke 1905–1962, over 50 years of gluten free diet. Gut.

[3] Bardella M.T., Elli L., Velio P., Fredella C., Prampolini L., Cesana B. (2007). Silent Celiac Disease Is Frequent in the Siblings of Newly Diagnosed Celiac Patients. Digestion.

[4] Megiorni F., Mora B., Bonamico M., Barbato M., Montuori M., Viola F., Trabace S., Mazzilli M.C. (2008). HLA-DQ and susceptibility to celiac disease: Evidence for gender differences and parent-of-origin effects. Am. J. Gastroenterol..

[5] Gujral N., Freeman H.J., Thomson A.B.R. (2012). Celiac disease: Prevalence, diagnosis, pathogenesis and treatment. World J. Gastroenterol..

[6] Tosi P., Gritsch C.S., He J., Shewry P.R. (2011). Distribution of gluten proteins in bread wheat (*Triticum aestivum*) grain. Ann. Bot..

[7] Corrao G., Corazza G.R., Bagnardi V., Brusco G., Ciacci C., Cottone M., Sategna Guidetti C., Usai P., Cesari P., Pelli M.A. (2001). Mortality in patients with coeliac disease and their relatives: A cohort study. Lancet.

[8] Mäki M., Collin P. (1997). Coeliac Disease. Lancet.

[9] Silvester J.A., Graff L.A., Rigaux L., Bernstein C.N., Leffler D.A., Kelly C.P., Walker J.R., Duerksen D.R. (2017). Symptoms of Functional Intestinal Disorders Are Common in Patients with Celiac Disease Following Transition to a Gluten-Free Diet. Dig. Dis. Sci..

[10] Stern E.K., Brenner D.M. (2018). Gut Microbiota-Based Therapies for Irritable Bowel Syndrome. Clin. Transl. Gastroenterol..

[11] Lovell R.M., Ford A.C. (2012). Global Prevalence of and Risk Factors for Irritable Bowel Syndrome: A Meta-analysis. Clin. Gastroenterol. Hepatol..

[12] Longstreth G., Thompson W.G., Chey W., Houghton L., Mearin F., Spiller R. (2006). Functional bowel disorders. Gastroenterology.

[13] Quigley E. (2018). The Gut-Brain Axis and the Microbiome: Clues to Pathophysiology and Opportunities for Novel Management Strategies in Irritable Bowel Syndrome (IBS). J. Clin. Med..

[14] Oswiecimska J., Szymlak A., Roczniak W., Girczys-Poledniok K., Kwiecien J. (2017). New insights into the pathogenesis and treatment of irritable bowel syndrome. Adv. Med. Sci..

[15] Drossman D.A. (2016). Functional gastrointestinal disorders: History, pathophysiology, clinical features, and Rome IV. Gastroenterology.

[16] Fass R., Longstreth G.F., Pimentel M., Fullerton S., Russak S.M., Chiou C.F., Reyes E., Crane P., Eisen G., McCarberg B. (2001). Evidence- and consensus-based practice guidelines for the diagnosis of irritable bowel syndrome. Arch. Intern. Med..

[17] Halmos E.P., Power V.A., Shepherd S.J., Gibson P.R., Muir J.G. (2014). A diet low in FODMAPs reduces symptoms of irritable bowel syndrome. Gastroenterology.

[18] Gibson P.R., Shepherd S.J. (2010). Evidence-based dietary management of functional gastrointestinal symptoms: The FODMAP approach. J. Gastroenterol. Hepatol..

[19] Staudacher H.M., Lomer M.C.E., Farquharson F.M., Louis P., Fava F., Franciosi E., Scholz M., Tuohy K.M., Lindsay J.O., Irving P.M. (2017). A Diet Low in FODMAPs Reduces Symptoms in Patients with Irritable Bowel Syndrome and a Probiotic Restores Bifidobacterium Species: A Randomized Controlled Trial. Gastroenterology.

[20] Nanayakkara W.S., Skidmore P.M., O’Brien L., Wilkinson T.J., Gearry R.B. (2016). Efficacy of the low FODMAP diet for treating irritable bowel syndrome: The evidence to date. Clin. Exp. Gastroenterol..

[21] Böhn L., Störsrud S., Liljebo T., Collin L., Lindfors P., Törnblom H., Simrén M. (2015). Diet Low in FODMAPs Reduces Symptoms of Irritable Bowel Syndrome as Well as Traditional Dietary Advice: A Randomized Controlled Trial. Gastroenterology.

[22] Pedersen N., Ankersen D.V., Felding M., Wachmann H., Végh Z., Molzen L., Burisch J., Andersen J.R., Munkholm P. (2017). Low-FODMAP diet reduces irritable bowel symptoms in patients with inflammatory bowel disease. World J. Gastroenterol..

[23] Cox S.R., Prince A.C., Myers C.E., Irving P.M., Lindsay J.O., Lomer M.C., Whelan K. (2017). Fermentable carbohydrates (FODMAPs) exacerbate functional gastrointestinal symptoms in patients with inflammatory bowel disease: A randomised, double-blind, placebo-controlled, cross-over, re-challenge trial. J. Crohn’s Colitis.

[24] Testa A., Imperatore N., Rispo A., Rea M., Tortora R., Nardone O.M., Lucci L., Accarino G., Caporaso N., Castiglione F. (2018). Beyond Irritable Bowel Syndrome: The Efficacy of the Low Fodmap Diet for Improving Symptoms in Inflammatory Bowel Diseases and Celiac Disease. Dig. Dis..

[25] Barrett J.S., Gibson P.R. (2010). Development and Validation of a Comprehensive Semi-Quantitative Food Frequency Questionnaire that Includes FODMAP Intake and Glycemic Index. J. Am. Diet. Assoc..

[26] Wilson B., Whelan K. (2017). Prebiotic inulin-type fructans and galacto-oligosaccharides: Definition, specificity, function, and application in gastrointestinal disorders. J. Gastroenterol. Hepatol..

[27] Patel S., Goyal A. (2012). The current trends and future perspectives of prebiotics research: A review. 3 Biotech.

[28] Marcason W. (2012). What Is the FODMAP Diet?. J. Acad. Nutr. Diet..

[29] Dugum M., Barco K., Garg S. (2016). Managing irritable bowel syndrome: The low-FODMAP diet. Cleve. Clin. J. Med..

[30] Staudacher H.M., Irving P.M., Lomer M.C.E., Whelan K. (2014). Mechanisms and efficacy of dietary FODMAP restriction in IBS. Nat. Rev. Gastroenterol. Hepatol..

[31] Dickson I. (2018). High FODMAP diet induces LPS-derived intestinal inflammation and visceral hypersensitivity. Nat. Rev. Gastroenterol. Amp. Hepatol..

[32] Zhou S.Y., Gillilland M., Wu X., Leelasinjaroen P., Zhang G., Zhou H., Ye B., Lu Y., Owyang C. (2018). FODMAP diet modulates visceral nociception by lipopolysaccharide-mediated intestinal inflammation and barrier dysfunction. J. Clin. Investig..

[33] Leffler D.A., Dennis M., Edwards George J.B., Jamma S., Magge S., Cook E.F., Schuppan D., Kelly C.P. (2009). A Simple Validated Gluten-Free Diet Adherence Survey for Adults With Celiac Disease. Clin. Gastroenterol. Hepatol..

[34] Mazzeo T., Roncoroni L., Lombardo V., Tomba C., Elli L., Sieri S., Grioni S., Bardella M.T., Agostoni C., Doneda L. (2016). Evaluation of a Modified Italian European Prospective Investigation into Cancer and Nutrition Food Frequency Questionnaire for Individuals with Celiac Disease. J. Acad. Nutr. Diet..

[35] Morreale F., Agnoli C., Roncoroni L., Sieri S., Lombardo V., Mazzeo T., Elli L., Bardella M.T., Agostoni C., Doneda L. (2018). Are the dietary habits of treated individuals with celiac disease adherent to a Mediterranean diet?. Nutr. Metab. Cardiovasc. Dis..

[36] Dall’Asta C., Scarlato A.P., Galaverna G., Brighenti F., Pellegrini N. (2012). Dietary exposure to fumonisins and evaluation of nutrient intake in a group of adult celiac patients on a gluten-free diet. Mol. Nutr. Food Res..

[37] (2008). European Institute of Oncology Food Composition Database for Epidemiological Studies in Italy. http://www.bda-ieo.it/.

[38] Mazzeo T., Cauzzi S., Brighenti F., Pellegrini N. (2015). The development of a composition database of gluten-free products. Public Health Nutr..

[39] Barratt S.M., Leeds J.S., Robinson K., Shah P.J., Lobo A.J., McAlindon M.E., Sanders D.S. (2011). Reflux and irritable bowel syndrome are negative predictors of quality of life in coeliac disease and inflammatory bowel disease. Eur. J. Gastroenterol. Hepatol..

[40] Sainsbury A., Sanders D.S., Ford A.C. (2013). Prevalence of irritable bowel syndrome-type symptoms in patients with celiac disease: A meta-analysis. Clin. Gastroenterol. Hepatol..

[41] O’Leary C., Wieneke P., Buckley S., O’Regan P., Cronin C.C., Quigley E.M.M., Shanahan F. (2002). Celiac disease and irritable bowel-type symptoms. Am. J. Gastroenterol..

[42] Hayes P., Corish C., O’Mahony E., Quigley E.M.M. (2014). A dietary survey of patients with irritable bowel syndrome. J. Hum. Nutr. Diet..

[43] De Giorgio R., Volta U., Gibson P.R. (2016). Sensitivity to wheat, gluten and FODMAPs in IBS: Facts or fiction?. Gut.

[44] Varney J., Barrett J., Scarlata K., Catsos P., Gibson P.R., Muir J.G. (2017). FODMAPs: Food composition, defining cutoff values and international application. J. Gastroenterol. Hepatol..

[45] Tuck C., Ly E., Bogatyrev A., Costetsou I., Gibson P., Barrett J., Muir J. (2018). Fermentable short chain carbohydrate (FODMAP) content of common plant-based foods and processed foods suitable for vegetarian- and vegan-based eating patterns. J. Hum. Nutr. Diet..

[46] Lordan R., Tsoupras A., Mitra B., Zabetakis I. (2018). Dairy Fats and Cardiovascular Disease: Do We Really Need to Be Concerned?. Foods.

[47] Lordan R., Zabetakis I. (2017). Invited review: The anti-inflammatory properties of dairy lipids. J. Dairy Sci..

[48] Lordan R., Tsoupras A., Zabetakis I. (2017). Phospholipids of animal and marine origin: Structure, function, and anti-inflammatory properties. Molecules.

[49] Bordoni A., Danesi F., Dardevet D., Dupont D., Fernandez A.S., Gille D., Nunes dos Santos C., Pinto P., Re R., Rémond D. (2017). Dairy products and inflammation: A review of the clinical evidence. Crit. Rev. Food Sci. Nutr..

[50] Gearry R.B., Irving P.M., Barrett J.S., Nathan D.M., Shepherd S.J., Gibson P.R. (2009). Reduction of dietary poorly absorbed short-chain carbohydrates (FODMAPs) improves abdominal symptoms in patients with inflammatory bowel disease-a pilot study. J. Crohn’s Colitis.

[51] Knight-Sepulveda K., Kais S., Santaolalla R., Abreu M.T. (2015). Diet and Inflammatory Bowel Disease. Gastroenterol. Hepatol..

[52] Shepherd S.J., Gibson P.R. (2006). Fructose Malabsorption and Symptoms of Irritable Bowel Syndrome: Guidelines for Effective Dietary Management. J. Am. Diet. Assoc..

[53] Maagaard L., Ankersen D.V., Vegh Z., Burisch J., Jensen L., Pedersen N., Munkholm P. (2016). Follow-up of patients with functional bowel symptoms treated with a low FODMAP diet. World J. Gastroenterol..

[54] De Roest R.H., Dobbs B.R., Chapman B.A., Batman B., O’Brien L.A., Leeper J.A., Hebblethwaite C.R., Gearry R.B. (2013). The low FODMAP diet improves gastrointestinal symptoms in patients with irritable bowel syndrome: A prospective study. Int. J. Clin. Pract..

[55] O’Keeffe M., Jansen C., Martin L., Williams M., Seamark L., Staudacher H.M., Irving P.M., Whelan K., Lomer M.C. (2018). Long-term impact of the low-FODMAP diet on gastrointestinal symptoms, dietary intake, patient acceptability, and healthcare utilization in irritable bowel syndrome. Neurogastroenterol. Motil..

[56] Staudacher H.M., Whelan K. (2016). Altered gastrointestinal microbiota in irritable bowel syndrome and its modification by diet: Probiotics, prebiotics and the low FODMAP diet. Proc. Nutr. Soc..

[57] Roncoroni L., Bascuñán K.A., Doneda L., Scricciolo A., Lombardo V., Branchi F., Ferretti F., Dell’osso B., Montanari V., Bardella M.T. (2018). A low FODMAP gluten-free diet improves functional gastrointestinal disorders and overall mental health of celiac disease patients: A randomized controlled trial. Nutrients.

